# Pathogenicity patterns in cytochrome P450 family

**DOI:** 10.1093/bioadv/vbaf231

**Published:** 2025-10-14

**Authors:** Anna Špačková, Nina Kadášová, Ivana Hutařová Vařeková, Karel Berka

**Affiliations:** Department of Physical Chemistry, Faculty of Science, Palacký University, Olomouc 771 46, Czech Republic; IT4Innovations, VSB—Technical University of Ostrava, Ostrava-Poruba 708 00, Czech Republic; Department of Physical Chemistry, Faculty of Science, Palacký University, Olomouc 771 46, Czech Republic; Department of Physical Chemistry, Faculty of Science, Palacký University, Olomouc 771 46, Czech Republic; CEITEC—Central European Institute of Technology, Masaryk University Brno, Brno 625 00, Czech Republic; Department of Physical Chemistry, Faculty of Science, Palacký University, Olomouc 771 46, Czech Republic

## Abstract

**Motivation:**

Cytochrome P450 proteins play a crucial role in human metabolism, ranging from hormone production to drug metabolism. While multiple commonly known variants have known effects on the individual cytochrome P450 protein performance, the pathogenicity information is usually experimentally limited to only a few mutations. Current pathogenicity prediction software enables the extension of the scope to virtually mutate all amino acids with all possible substitutional mutations. In this work, we do a comprehensive exploration that unveils pathogenicity patterns in the human cytochrome P450 family. Pathogenicity analysis was conducted across proteins using SIFT, AlphaMissense, and PrimateAI-3D algorithms.

**Results:**

Our findings indicate a progressive increase in pathogenicity along protein tunnels—identified via MOLE—toward the cofactor binding site, underscoring the essential role of cofactor interactions in enzymatic function. Notably, the integrity of tunnels and cofactor environment emerges as a critical factor, with even single amino acid alterations potentially disrupting molecular guidance to active sites. These insights highlight the fundamental role of structural pathways in preserving cytochrome P450 functionality, with implications for understanding disease-associated variants and drug metabolism.

**Availability and implementation:**

Data and source code can be found at https://github.com/annaspac/P450_pathogenicity_codes.

## 1 Introduction

The human cytochrome P450 (CYP) family comprises 57 genes, with diverse roles in drug metabolism, processing of foreign chemicals, synthesis and metabolism of vitamin D3 (Kitanaka *et al.* 1998, Sawada *et al.* 1999, Mohamed *et al.* 2024), calcium and phosphorus homeostasis (Tang *et al.* 2012). It emphasizes their pivotal role in maintaining physiological integrity (Nebert *et al.* 2013). The physiological importance of CYP enzymes is further underscored by the growing body of evidence linking specific CYP polymorphisms and rare mutations to various non-cancer diseases. For example, loss-of-function mutations in CYP1B1 are a well-established cause of primary congenital glaucoma, a rare but severe developmental eye disorder (Stoilov *et al.* 1997, Bhattacharjee *et al.* 2008). In reproductive health, CYP2C19 poor metabolizer alleles have been linked to a higher susceptibility to endometriosis, likely due to altered hormone metabolism (Painter *et al.* 2014). In hepatic disease, the promoter variant rs2031920 in CYP2E1 has been suggested to influence the risk of alcoholic liver disease by modulating enzyme expression (Zeng *et al.* 2013). Similarly, the CYP2J2 variant rs890293 has been associated with cardiovascular complications, possibly through reduced EET formation (Berlin *et al.* 2011). These genotype–phenotype associations highlight the significant impact of CYP genetic diversity on human health.

A critical feature influencing the function of enzymes like CYPs is the presence of tunnels within their structures. These tunnels, found in various biomacromolecules including proteins and nucleic acids, serve as pathways that facilitate the transport of substrates, products, and cofactors between internal active sites and the external cellular environment (Huang *et al.* 2001, Pravda *et al.* 2014). The presence of a tunnel streamlines the selection of substances that are allowed to enter specific regions within the protein amidst the complex mix of molecules in the cell, particularly toward its active sites (Marques *et al.* 2017). Tunnels represent significant structural features that may contribute to the control of enzymatic functions and other biological processes (Ljungdahl *et al.* 2023). While such structural elements play a key role in modulating enzyme activity, enzymatic function also critically depends on the presence of cofactors. Acting as redox carriers in biosynthetic and catabolic pathways, cofactors enable efficient energy transfer within the cell (Strushkevich *et al.* 2010). Most cofactors bind non-covalently to proteins, though some form covalent attachments, influencing enzyme stability and activity (Stevens *et al.* 2005).

Given the central role of tunnels and cofactors in enzyme function, regions surrounding these structural features may be particularly sensitive to disruption. Recent advances in genome sequencing revealed significant genetic diversity in human populations (Karczewski *et al.* 2020). Such mutations can alter protein amino acid sequences and structure, with pathogenic variants impairing protein function and benign variants causing minimal disruption (Wang *et al.* 2013). Significantly higher numbers of pathogenic mutations were in buried residues in CYP structures compared to exposed residues (Mondal *et al.* 2025). In contrast, our study focuses specifically on amino acid substitutions located in the vicinity of protein tunnels and cofactors—structural features essential for substrate transport and catalytic function. We hypothesize that variants near these regions are particularly likely to disrupt protein activity, and thus more prone to be pathogenic. To test this, we compare predicted pathogenicity scores across the whole protein sequence and in subsets of residues near tunnels and cofactors.

## 2 Methods

### 2.1 Human CYP structure dataset and MOLE tunnel computation

To identify human CYP enzymes, the UniProt database (UniProt Consortium 2023) was queried for proteins belonging to the “cytochrome P450 family,” restricted to the organism *Homo sapiens*, and limited to entries with reviewed status. This yielded 60 canonical human CYP proteins, whose UniProt IDs were subsequently used to search the AlphaFill database (Hekkelman *et al.* 2023), which provides a consistent dataset for all human CYP protein model structures enriched with heme cofactor (HEM). Since less than half of human CYPs have any experimental structure (precisely 27), and our previous benchmark on CYP2D6 (Špačková et al. 2024) showed that AlphaFill models reliably reproduce known tunnels positions seen in crystallographic structures, we have included only full-length AlphaFill models for all CYPs to have a consistent dataset.

Protein tunnels in these structures were analysed using the MOLE 2.5.24.6.8 version of the MOLE algorithm (Sehnal *et al.* 2013, Pravda *et al.* 2018), enabling quantification of tunnel properties and analysis of the surrounding amino acid environment. Interestingly, four proteins in the dataset were reported to lack any detectable tunnels under the default MOLE settings ProbeRadius = 5, InteriorThreshold = 1.1, IgnoreHETAtoms = 1, SurfaceCoverRadius = 10, OriginRadius = 5, BottleneckRadius = 1.2, BottleneckTolerance = 3, MaxTunnelSimilarity = 0.7, and the HEM cofactor was used as the starting point for tunnel detection. Using this setup, we have been able to calculate tunnels in 56 structures and we further use a reduced dataset of proteins that contain tunnels.

### 2.2 Pathogenicity of amino acids

Pathogenicity scores were computed for the dataset of 56 CYP protein structures using the AlphaMissense algorithm (Cheng *et al.* 2023), search based on UniProt ID. Subsequently, the SIFT algorithm (Ng and Henikoff 2003) was applied to the same dataset, using protein sequences in FASTA format. For each residue, pathogenicity scores were calculated for all available non-synonymous amino acid substitutions and then averaged. Average pathogenicity values were computed per protein using both AlphaMissense and SIFT methods. Additionally, we mapped gene names to their Ensembl canonical transcripts and searched for corresponding variant pathogenicity scores for the subset of CYP genes available in the PrimateAI-3D (Gao *et al.* 2023) dataset. Only 41 CYPs had data available in the PrimateAI-3D dataset, and average pathogenicity scores were calculated only for these.

AlphaMissense, SIFT, and PrimateAI-3D are widely used tools for predicting variant pathogenicity but differ in methodology and sensitivity. AlphaMissense is a deep learning-based method based on fine-tuned AlphaFold protein structural modelling and a protein language model trained on population frequency data and masked multiple sequence alignment. SIFT relies on sequence conservation with an inverse scoring system, often producing more conservative predictions. PrimateAI-3D integrates a 3D-convolutional neural network, incorporating evolutionary conservation and protein 3D structure from AlphaFoldDB, providing a balanced prediction that captures spatial protein context better than SIFT but usually with less pronounced scores than AlphaMissense (Ljungdahl *et al.* 2023). Together, these tools complement each other by combining diverse approaches to the computation of pathogenicity (Supplementary Table S1).

Further analysis focused on amino acids near tunnels identified by the MOLE algorithm, with their respective pathogenicity values determined. Similarly, for amino acids around cofactors, the nearest amino acids were identified for each cofactor atom. Tunnel‐lining residues were defined from MOLE results by sampling the tunnel central line at regular points and assigning the five closest protein atoms (minimum one, maximum five residues per point). The union of all such residues formed the tunnel set. Cofactor‐proximal residues were analogously defined as the five closest protein atoms for each cofactor atom, with the final set taken as the non-redundant union. The average pathogenicity was computed from the 20 representatives of all available non-synonymous substitutions. This comprehensive approach provided insights into the average pathogenicity for entire proteins, the vicinity of tunnels, and the regions around cofactors.

All results were processed and obtained using codes programmed in Python 3.9.13, utilizing libraries such as os, json, pandas, seaborn, and matplotlib.pyplot.

### 2.3 2DProts

2DProts (Hutařová Vařeková *et al.* 2021) is a software tool designed for the visualization of protein secondary structure through two-dimensional diagrams. It forms part of the CATH protein family database (Sillitoe *et al.* 2021).

## 3 Results

**Figure 1.**
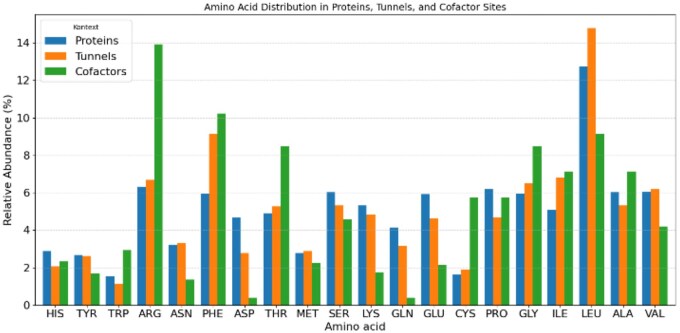
Amino acid distribution in proteins, tunnels, and cofactor sites. For protein structures and tunnels, the highest percentage representation is for leucine. However, this changes when looking around cofactors, where the highest relative frequency is observed for arginine. Compared to whole proteins, phenylalanine, isoleucine, and leucine show a higher relative frequency in tunnels. An increase in relative frequency around cofactors compared to tunnels is also observed for arginine, phenylalanine, threonine, and glycine.

In this study, we analysed 56 CYP protein structures, where we found tunnels, with a focus on identifying protein tunnels and examining the regions surrounding cofactors. By comparing the amino acid composition in these functionally important areas, we observed notable differences in the relative abundance of specific residues across whole CYP proteins, their tunnels, and around cofactors (Fig. 1).

**Figure 2.**
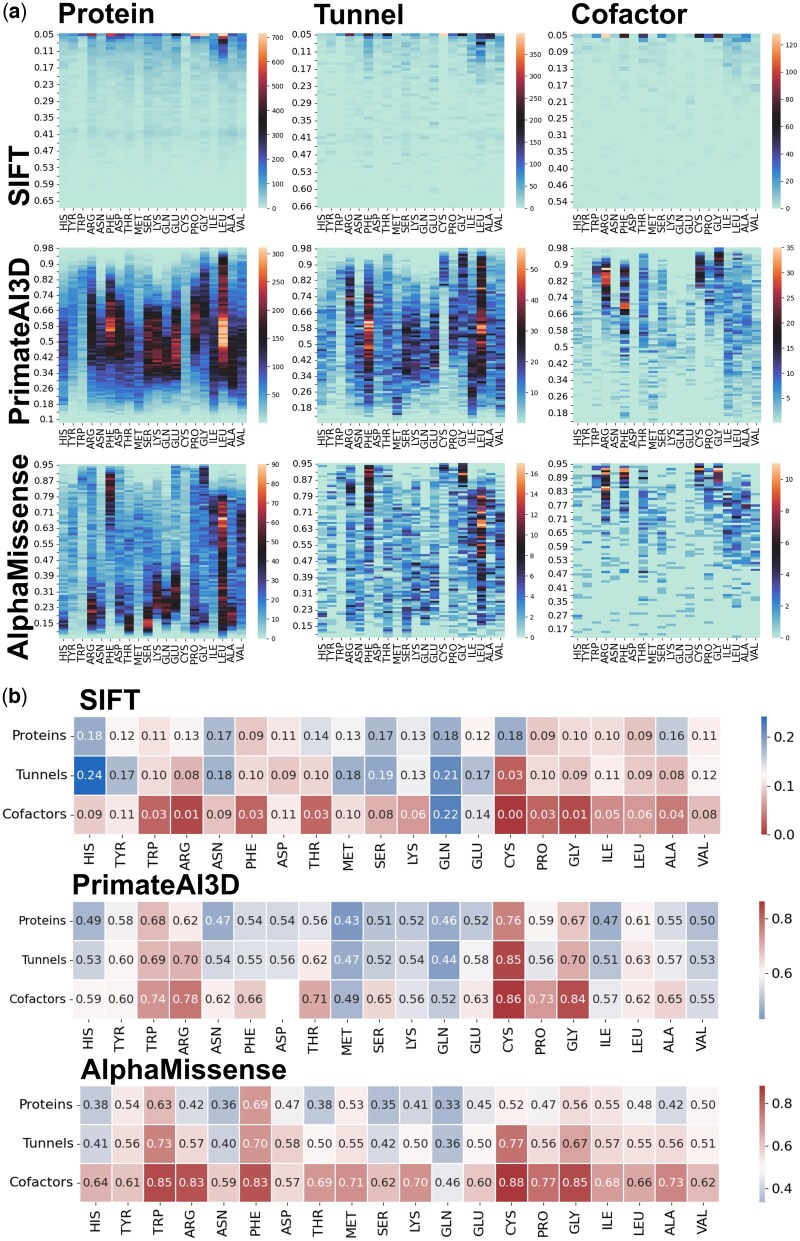
(a) Pathogenicity in human cytochrome P450, around tunnels, and cofactors assessed using AlphaMissense, SIFT, and PrimateAI-3D. Heatmap illustrates the frequency of occurrence of specific amino acids with their corresponding pathogenicity values. AlphaMissense and PrimateAI-3D results show a range of pathogenicity for entire proteins, from 0 (benign) to 1 (pathogenic), with residues around tunnels mostly at higher values. This pattern intensifies around cofactors. The SIFT algorithm maintains this trend, but most amino acids are designated as pathogenic, particularly around the value of 0.05 in this case, from 0 (pathogenic) to 1 (benign). (b) Comparison of average pathogenicity in entire proteins, around tunnels, and near cofactors in cytochromes P450. In proteins, phenylalanine and tryptophan exhibit the highest pathogenicity, followed by glycine. Around tunnels, the trend changes, with cysteine showing the highest pathogenicity, followed by tryptophan and phenylalanine with consistently high pathogenicity. Near cofactors, cysteine, glycine, tryptophan, arginine, and phenylalanine are the most pathogenic. Overall, the trend persists, with average pathogenicity increasing from proteins, through tunnels, to cofactors, for each represented amino acid.

Building on the structural differences identified around tunnels and cofactors, we further examined the pathogenicity profiles of individual residues located in these regions. After categorizing pathogenicity based on individual residues, there is an observed increased occurrence of amino acids with higher pathogenicity values for residues found around tunnels and cofactors. The occurrence of values representing benign mutations is significantly suppressed in protein-tunnel and tunnel-cofactor scenarios. This holds true for the distribution of amino acids primarily in AlphaMissense, and partially in SIFT and PrimateAI-3D calculations (Fig. 2).

The scale of pathogenicity differs between SIFT and AlphaMissense, and PrimateAI-3D algorithms. AlphaMissense and PrimateAI-3D ranges from zero, representing benign substitutions, to one, representing pathogenic substitutions. The SIFT algorithm uses a reversed scoring scale compared to other tools.

Values from AlphaMissense observed in CYP proteins are distributed more or less regularly. Values up to 0.35 are considered benign, values between 0.35 and 0.65 are considered uncertain, and values above 0.65 are considered pathogenic. However, patterns emerge showing an increased occurrence of amino acids with certain pathogenicity. Elevated occurrences above a value of 0.5, combined with a large number of such pathogenic amino acids, are observed for phenylalanine, glycine, isoleucine, leucine, and valine across the entire protein. Pathogenic amino acids are enriched near tunnels and cofactors, whereas benign variants are largely absent in these regions (Fig. 2).

Pathogenicity scores from PrimateAI-3D span the full range from 0 to 1, yet show a clear separation in their distributions: benign mutations have a mean score of 0.47, while pathogenic mutations cluster around a higher mean of 0.77. An increased occurrence of leucine, phenylalanine, and arginine can be observed in proteins. In tunnels, leucine, glycine, phenylalanine, and arginine are more represented. Around cofactors, glycine, cysteine, phenylalanine, arginine, and tryptophan are enriched (Fig. 2a).

Conversely, in the SIFT algorithm, the scale is inverted, with most amino acids being designated as highly pathogenic. However, a reduced presence of less pathogenic amino acids is observed around tunnels and, to a greater extent, around cofactors, where they are almost absent (Fig. 2a). In entire protein structures, amino acids with the highest pathogenicity, such as leucine, glycine, proline, phenylalanine, and tryptophan, are most abundant. Notably, around cofactors, glycine predominates in pathogenicity, followed by arginine, phenylalanine, and threonine. Additionally, arginine, phenylalanine, glycine, cysteine, threonine, and proline are prominently represented among cofactors. Alanine, leucine, and isoleucine are also present, albeit to a lesser extent.

A comparison of the average pathogenicity of individual amino acids was conducted. The findings from AlphaMissense conform to the protein-tunnel and tunnel-cofactor patterns, with one exception: aspartic acid, where the difference is only 0.01. Among the most pathogenic amino acids in the entire protein were phenylalanine, tryptophan, glycine, isoleucine, and tyrosine. Around tunnels, the order of the most pathogenic amino acids averaged cysteine, tryptophan, phenylalanine, and glycine. Amino acids in the vicinity of cofactors, including cysteine, tryptophan, glycine, phenylalanine, and arginine, exhibited some of the highest levels of pathogenicity (Fig. 2a). Overall, if an amino acid ranks among the most pathogenic in one of the groups whole protein, tunnel vicinity, or cofactor vicinity, it tends to display higher pathogenicity in the other groups as well.

The SIFT results maintain the pattern for 10 amino acids. Among the amino acids with the highest average pathogenicity in whole proteins are leucine, proline, phenylalanine, isoleucine, glycine, and tryptophan. In the tunnel region, cysteine predominates in pathogenicity, followed by arginine, alanine, leucine, glycine, and aspartic acid. Within the cofactor vicinity, the most pathogenic amino acids are cysteine, glycine, arginine, phenylalanine, proline, tryptophan, threonine, and alanine (Fig. 2b).

PrimateAI-3D analysis revealed that glycine, cysteine, arginine, and tryptophan are consistently among the most pathogenic residues across all examined contexts: whole protein structures, internal tunnels, and cofactor-binding regions. Interestingly, asparagine was not observed at all in the vicinity of cofactors in any of the 41 analysed transcripts. Three amino acids do not follow the general increasing trend, one of which was missing a value for the region surrounding the cofactor (Fig. 2b).

All three tools agree on the amino acids phenylalanine, isoleucine, glycine, and tryptophan in whole proteins, cysteine and glycine around tunnels, and cysteine, glycine, phenylalanine, and tryptophan around cofactors (Fig. 2b). The number of individual amino acids around the tunnels varied significantly, with leucine and phenylalanine being the most abundant, comprising around 15%. Approximately 7% representation was observed for isoleucine and arginine, while valine accounted for 6%. The least represented amino acids around the tunnels were aspartic acid and histidine.

AlphaMissense adheres to the average pathogenicity pattern in the protein, tunnels, and cofactors environment at 93.1%, while SIFT determination adheres to the pattern only at 45.0% and PrimateAI-3D only at 63.4%. For more accurate results, predictions can be made using the AlphaMissense algorithm, which is among the most effective pathogenicity prediction algorithms. The average pathogenicity value for proteins calculated using SIFT ranges from 0.08 to 0.20, while for AlphaMissense, it spans from 0.29 to 0.91, and in the case of PrimateAI-3D, it ranges from 0.47 to 0.64. The wider range used with AlphaMissense ensures a more accurate determination of pathogenicity.

Examining the phylogenetic tree, the average pathogenicity value correlates with relatedness in the phylogenetic tree, especially among proteins within the same branch in SIFT results, where the algorithm is based on sequences and their conservation. It is fascinating to observe that individual branches adhere to pathogenicity within certain ranges, notably in learning the pathogenicity of the entire protein, where a similarity in pathogenicity is preserved within the same branches of the phylogenetic tree (Supplementary Figs S1, S2). Near cofactors, this value remains largely unchanged except for two branches, which differ significantly.

In AlphaMissense, there is a certain similarity in the values of pathogenicity, but also occasional exceptions, which typically consist of three distinct values in the columns representing pathogenicity for the entire protein, tunnels, and around cofactors. A similar pattern is observed with the results from PrimateAI-3D.

**Figure 3.**
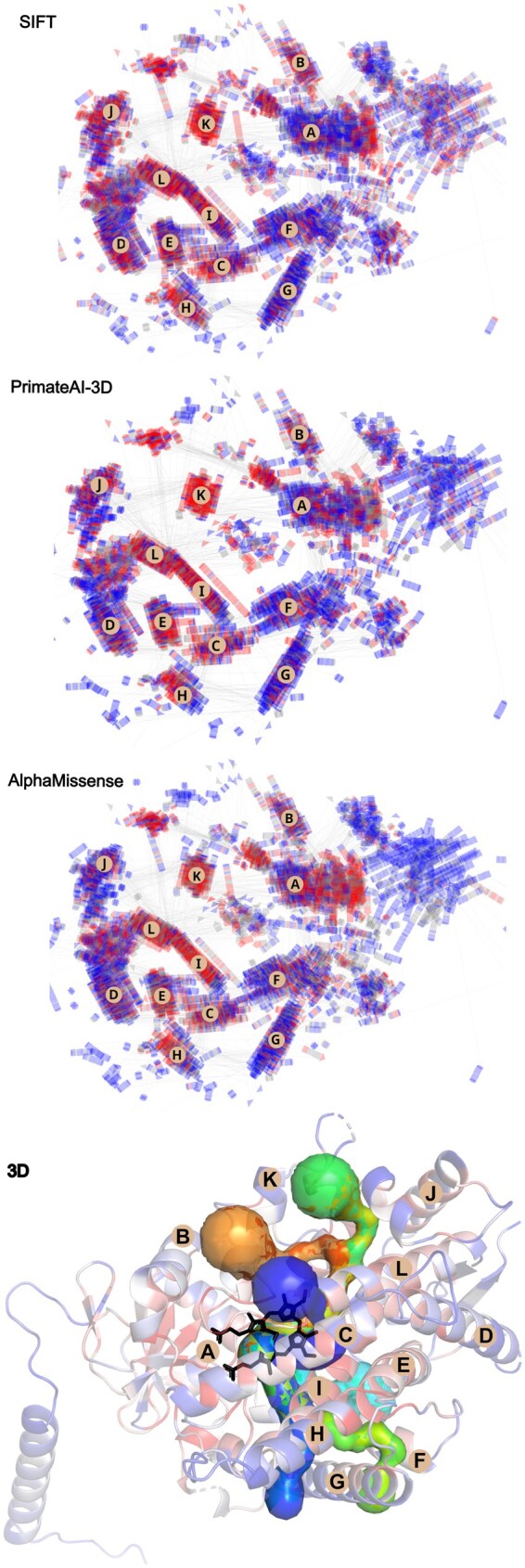
Overlaid two-dimensional (2D) representations of CYP proteins generated with 2DProts, displaying SIFT, PrimateAI-3D, and AlphaMissense-predicted pathogenicity scores. Redder areas indicate regions with higher pathogenicity, potentially indicating clusters of pathogenic mutations within specific regions of the proteins. In the structure, tunnels are depicted across all proteins using 2DProts. The secondary structure A shows the highest pathogenicity in AlphaMissense. Secondary structures K, I, L, E, and H exhibit increased pathogenicity across all tools—SIFT, PrimateAI-3D, and AlphaMissense. For comparison of 2D and three-dimensional (3D) representations, the structure of P04798 is shown together with the HEM group and tunnels. The 3D structure is color-coded on a blue-red scale indicating pathogenicity scores predicted by AlphaMissense, and the secondary structure elements are labeled consistently with those in the 2D representation according to SecStrAnnotator (https://sestra.ncbr.muni.cz/) (Midlik *et al.* 2021).

Pathogenicity predictions obtained from SIFT, PrimateAI-3D, and AlphaMissense consistently highlight structurally conserved regions of CYP proteins as hotspots for potentially deleterious variants. In particular, helices L, I, K, and H show an accumulation of residues with high predicted pathogenicity, whereas other regions are enriched in variants predicted to be benign. This convergence across independent methods underscores the functional importance of these secondary structures in maintaining CYP stability and activity (Fig. 3).

**Figure 4.**
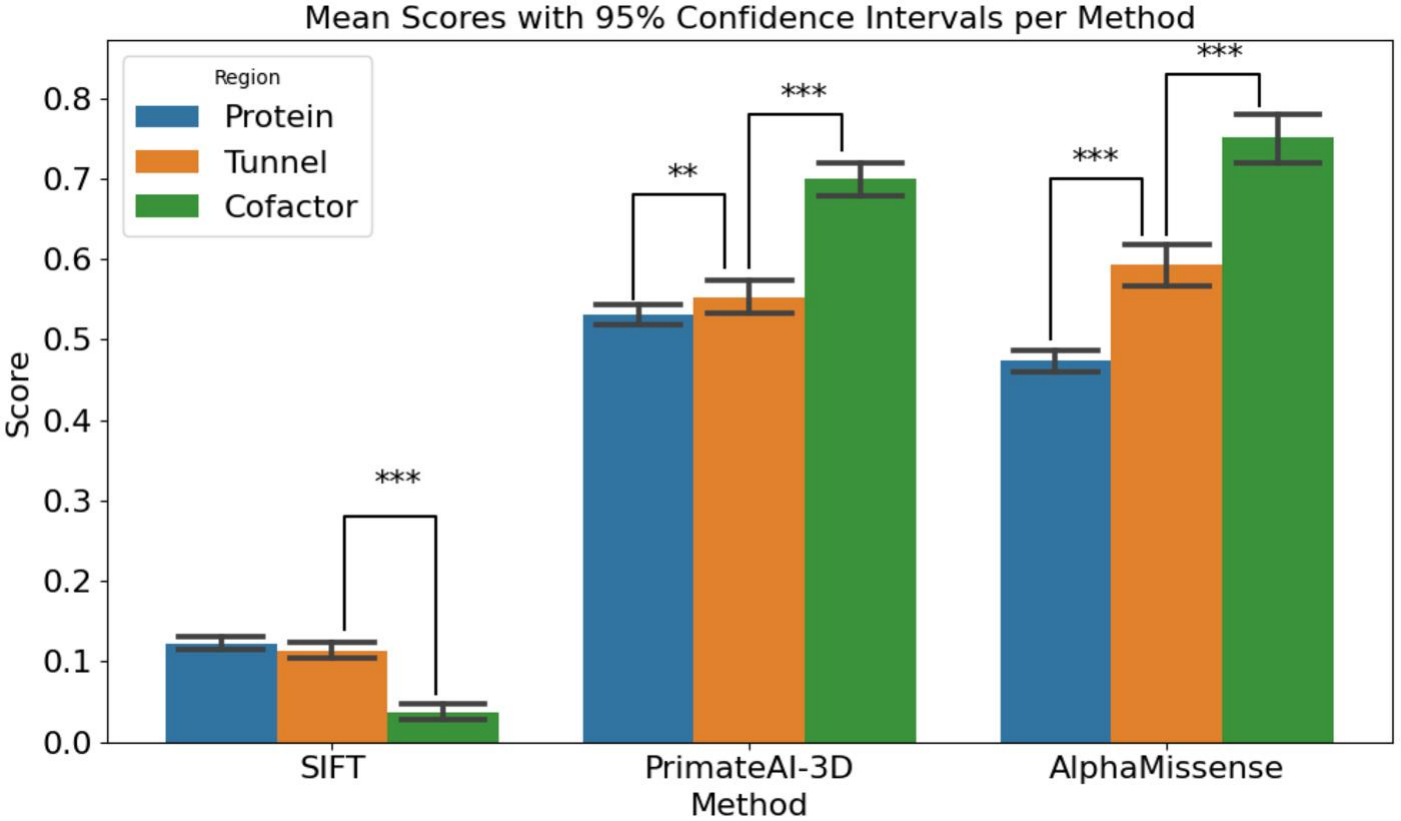
Average pathogenicity scores from SIFT, PrimateAI-3D, and AlphaMissense are shown for variants located in three protein regions: the general protein structure (Protein), tunnels (Tunnel), and cofactor-binding sites (Cofactor). Bars represent mean scores with 95% confidence intervals. Asterisks above the bars denote statistical significance based on paired *t*-tests comparing Protein versus Tunnel and Tunnel versus Cofactor regions (*P < .05; **P < .005; ***P < .0005). All three methods consistently indicate an increase in pathogenicity from the general protein background toward tunnel and cofactor regions, with the most pronounced differences observed in AlphaMissense. Although SIFT uses an inverse scoring system, where lower values indicate higher pathogenicity, it exhibits the same trend. These findings emphasize that functionally critical regions, such as tunnels and cofactor-binding sites, are more sensitive to substitution mutation.

To evaluate whether variants located in tunnels and cofactor-binding regions are associated with higher predicted pathogenicity, we compared average scores from SIFT, PrimateAI-3D, and AlphaMissense across three structural categories: general protein regions, tunnel-lining residues, and cofactor-binding sites. As shown in Fig. 4, all three predictors revealed a statistically significant increase in pathogenicity from general protein areas toward tunnels and cofactor-binding regions. Paired t-tests confirmed statistically significant differences in most comparisons, with the exception of the SIFT score between protein and tunnel regions, where the *P* value (.055) was just above the conventional threshold for significance. Despite SIFT’s inverse scoring scale, where lower values indicate higher pathogenicity, the overall trend was consistent across all tools. These findings support the idea that mutations in structurally and functionally important regions are more likely to have a damaging effect.

## 4 Discussion

Cytochromes P450 are essential for the proper functioning of the human body, appearing in several physiological processes associated with various diseases. CYP proteins contain the HEM cofactor.

The HEM-binding loop, located on the proximal side before the L helix, contains the hallmark CYP consensus sequence (Phe-X-X-Gly-X-Arg-X-Cys-X-Gly), including the absolutely conserved cysteine that serves as the axial thiolate ligand to the HEM iron (Werck-Reichhart and Feyereisen 2000), which makes this amino acid the most critical one. This covalent-like coordination is indispensable for electron transfer and catalytic activation of oxygen, and substitution in this part abolishes the activity of the CYP enzyme (Guengerich 2001, Denisov *et al.* 2005).

Glycines can be found in CYPs mostly in the conserved motifs and tight turns. As glycines lack the side chain, they confer flexibility and permit the sharp backbone angles necessary to accommodate the HEM prosthetic group. Therefore, steric constraints and destabilization of the fold can occur after mutation on glycine spots (Li *et al.* 2008).

Phenylalanine can be found as part of the PERF motif, which is a part of the electron transport chain to the active site (Fang *et al.* 2024). Phenylalanines also contribute to the hydrophobic packing of helices surrounding the active site. Moreover, phenylalanine and arginine are described as gatekeeping amino acid residues (Šrejber *et al.* 2018). For instance, the substrate channel is shaped and stabilized by phenylalanine’s aromatic rings; its replacement by polar residues would disrupt hydrophobic contacts, compromising active site geometry (Li *et al.* 2008, Hsu *et al.* 2017).

Highly conserved threonine is located in the central region of the I helix on the distal side. It harbours another CYP signature motif (Ala/Gly-Gly-X-Asp/Glu-Thr-Thr/Ser), which forms part of the proton transfer groove and is essential for maintaining catalytic activity over time and oxygen activation itself (Werck-Reichhard and Feyereisen 2000). The I helix plays a crucial role in the catalytic cycle, primarily in dioxygen activation. The mechanism prevents rapid auto-inactivation during catalytic turnover and protects against oxidative damage by stabilizing (Yoshigae *et al.* 2013). Its importance has also been demonstrated in previous studies, where its replacement by another amino acid led to increased CYP 2E1 inactivation and HEM destruction (Blobaum *et al.* 2004, Yoshigae *et al.* 2013).

Finally, arginine residues in CYP have been described as forming an interaction with NADPH-CYP reductase (CYPOR). CYPOR enzyme is essential for electron transfer during the catalytic cycle. For this protein-protein interaction, showing residues of Arg of strong importance, as well as residues of Lys, influencing the overall catalytic function of the enzyme (Shen and Strobel 1993). The absolutely conserved Glu-X-X-Arg motif in helix K, also on the proximal side, likely plays a crucial role in stabilizing the core structure.

Overall, this conserved structure and sequence, particularly the presence of amino acids such as Thr, Arg, Phe, Cys, and Gly within the key motifs of the HEM-binding loop and helices K, I, and L, may explain why these specific residues were identified as pathogenic in our study. It also likely accounts for why the secondary structures of helices K, I, and L are predicted as highly pathogenic regions in the 2DProts analysis. The enrichment of these amino acids in high-pathogenicity regions can also reflect their key structural roles within the substrate and product tunnels.

The calculation of protein tunnels using the MOLE tool provides information about the tunnels surrounding residues and their physicochemical properties, which subsequently influences the types of molecules that can enter the tunnels and react with the buried active site. For example, variants A330F and V364M, which occurred in tunnels of the CYP1B1 protein, are associated with primary congenital glaucoma, anterior segment dysgenesis type 6, and congenital glaucoma (ClinVar Accession VCV002203049; ClinVar VCV002203049 2025); additionally, the V364M variant has been linked to Glaucoma 3A (ClinVar Accession VCV001339668; ClinVar VCV001339668 2025). These mutations highlight the critical role of CYP1B1 in anterior segment development and intraocular pressure regulation.

## 5 Conclusions

Studying the pathogenicity of different parts of CYPs provides information about their importance. The study examines the pathogenicity of individual amino acids present in proteins. One of the key findings in this study is the increasing pathogenicity in protein-tunnel and tunnel-cofactor pairs (Fig. 4). This increase suggests that amino acid substitutions across the entire protein are not as severe as those occurring near tunnels and even more so in the vicinity of cofactors. This substitution can affect the physicochemical properties at the sites where transport and reactions with the active site occur in the protein.

The comparison of results from the SIFT, AlphaMissense, and PrimateAI-3D algorithms provides information on the reliability and accuracy of pathogenicity prediction outcomes. While algorithms agree on certain amino acids with high pathogenicity, such as phenylalanine, glycine, cysteine, and tryptophan, in the entire protein as well as in the regions of tunnels and cofactors, there are noticeable differences. Pathogenicity values range from 0 to 1, with AlphaMissense and PrimateAI-3D designating 1 as pathogenic and 0 as benign, whereas with the SIFT algorithm, it is the opposite. Results are distributed across the full scale in AlphaMissense, almost the same as PrimateAI-3D, but with SIFT, pathogenicity for most amino acids is determined up to a value of 0.05.

The phylogenetic tree within the cytochrome P450 family shows a certain correlation with pathogenicity patterns in the case of related protein sequences. Preservation of pathogenicity in specific branches of the tree is particularly evident in the results of the SIFT algorithm, which may be attributed to how the algorithm determines pathogenicity and its close association with multiple sequence alignment of proteins (Sim *et al.* 2012). Overall, the preservation of pathogenicity in branches underscores the functional significance of related sequences.

The analysis of amino acid occurrence around tunnels and cofactors reveals an increased presence of pathogenicity and specific amino acids, further emphasizing the importance of the integrity of tunnels for transporting small molecules to the active site.

## Supplementary Material

vbaf231_Supplementary_Data

## Data Availability

Data and source code can be found at https://github.com/annaspac/P450_pathogenicity_codes.
