# Correction to: Pathogenicity patterns in cytochrome P450 family

**DOI:** 10.1093/bioadv/vbag161

**Published:** 2026-07-02

This is a correction to: Anna Špačková, Nina Kadášová, Ivana Hutařová Vařeková, Karel Berka, Pathogenicity patterns in cytochrome P450 family, *Bioinformatics Advances*, Volume 5, Issue 1, 2025, vbaf231, https://doi.org/10.1093/bioadv/vbaf231

In the originally published version of this manuscript, incorrect DOIs were supplied for references 11, 19, 23, 26, 27, 35, and 40. These have been corrected as follows:
