# Supplementary material for: Pathogenicity patterns in cytochrome P450 family
Source: Bioinform Adv. 2025 Oct 14;5(1):vbaf231. doi: 10.1093/bioadv/vbaf231 (PMC12534787; doi:10.1093/bioadv/vbaf231)
Supplement: vbaf231_Supplementary_Data [file vbaf231_supplementary_data.docx]

Supplemental information for

**Pathogenicity Patterns in Cytochrome P450 Family**

Anna Špačková^1,2,*^ , Nina Kadášová^1^, Ivana Hutařová Vařeková^1,3,4^, Karel Berka^1,*^

^1^ Department of Physical Chemistry, Faculty of Science, Palacký University, tř. 17. listopadu 12, 771 46 Olomouc, Czech Republic, ^2^ IT4Innovations, VSB – Technical University of Ostrava, 17. listopadu 2172/15, 708 00 Ostrava-Poruba, Czech Republic, ^3^ CEITEC – Central European Institute of Technology, Masaryk University Brno, Kamenice 5, 625 00 Brno, Czech Republic, ^4^ National Centre for Biomolecular Research, Faculty of Science, Masaryk University Brno, Kamenice 5, 625 00 Brno, Czech Republic

**Table S1** - Comparison of methodological differences between individual pathogenicity predictors

| **Feature** | **AlphaMissense** | **SIFT** | **PrimateAI-3D** |
| --- | --- | --- | --- |
| **Principle** | Deep learning (fine-tuned protein structural modelling and a pLM trained on population frequency data and masked MSA). | Evolutionary conservation from MSA. | Deep NN using 3D protein structure from AlphaFoldDB + primate evolutionary data. |
| **Data basis** | Large variant datasets and fine-tuned AlphaFold models. | Cross-species sequence alignments. | Human and primate genomes, structural modelling. |
| **Output** | Pathogenicity probability (0–1), higher = more pathogenic. | Score 0–1, pathogenic if <0.05. | Pathogenicity probability (0–1), higher = more pathogenic. |
| **Strengths** | Structural context from AlphaFold, broad coverage, good generalisation. Best on ClinVar benchmarks. | Simple, fast, interpretable; widely used. | Incorporates structural context, higher accuracy on clinically relevant variants. |
| **Limitations** | Dependent on AlphaFold model quality, weaker for rare proteins. Available only for humans. | No structural info - less accurate for poorly conserved or short proteins. | Dependent on static AlphaFoldDB model quality, trained on a limited species - potential bias beyond humans, computationally intensive. |


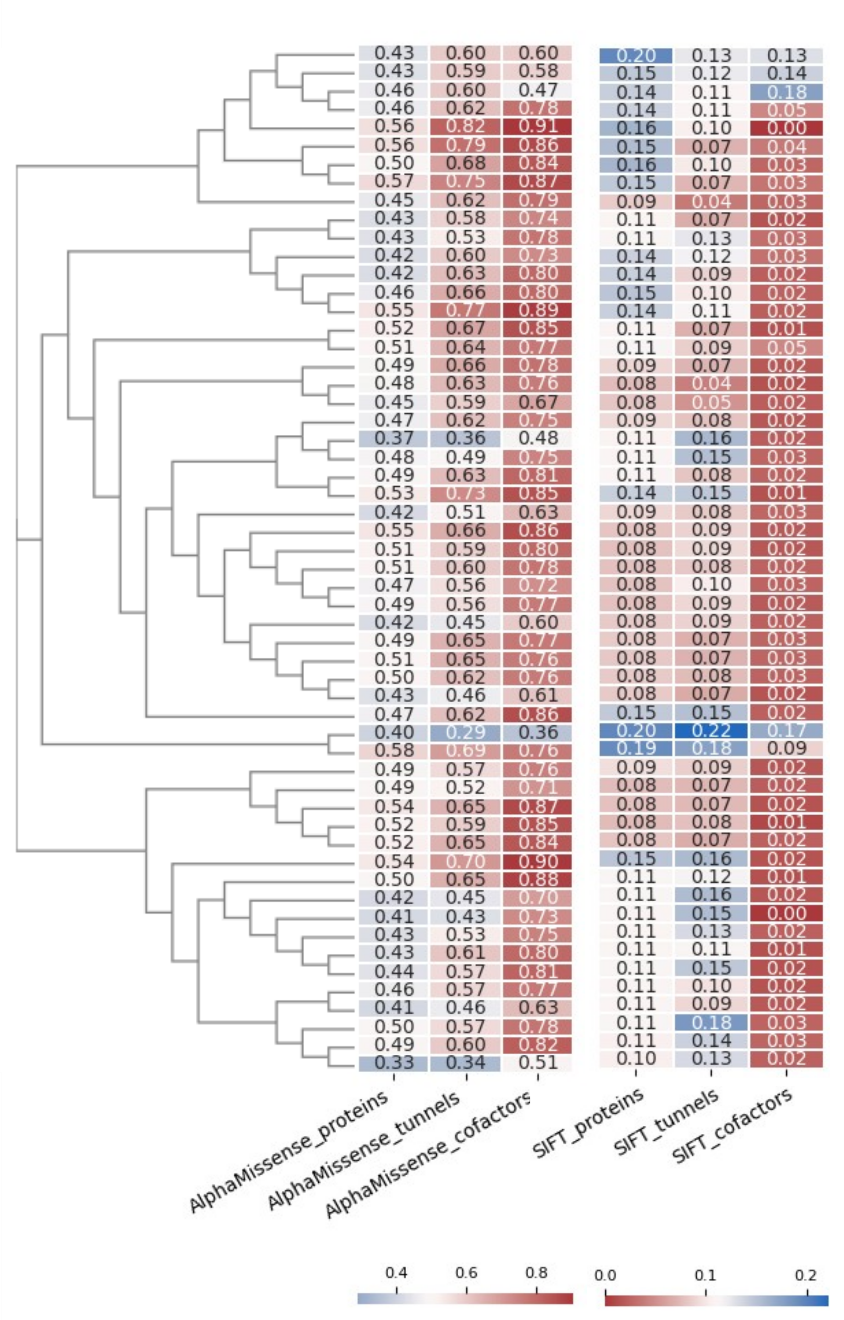


**Fig. S1** - Comparing the average pathogenicity in P450 cytochromes, focusing on entire proteins, tunnels, and cofactor surroundings, with protein phylogenetics. Most of the protein follows the pattern of increasing average pathogenicity from the entire protein through tunnels to cofactors. For AlphaMissense results, four do not follow the pattern of increasing pathogenicity from protein, through tunnels, to cofactors. Two do not exhibit increased pathogenicity around tunnels compared to the entire protein, and in one case, there is no higher pathogenicity for the entire protein compared to the cofactor surroundings. The average pathogenicity for all but two is higher around tunnels compared to the entire protein. With two exceptions, the average pathogenicity increases around cofactors compared to the average pathogenicity in tunnels. With SIFT, the preservation of pathogenicity is clearly visible in individual branches of relatedness for the average pathogenicity of the protein.


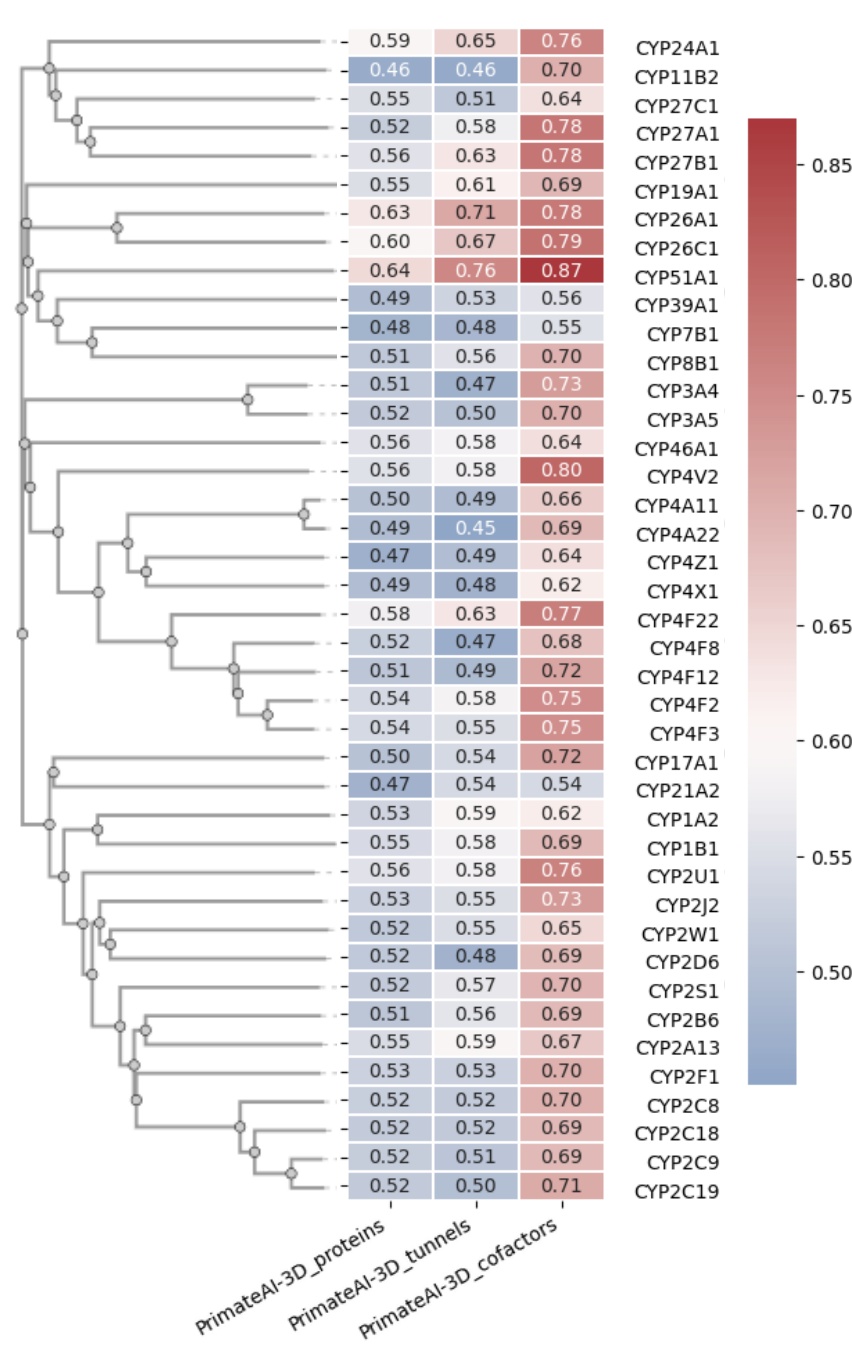


**Fig. S2 -** The phylogenetic distribution of PrimateAI-3D scores reflects conserved patterns of functional constraint, particularly in regions mediating substrate access.
